# Causal beliefs about obesity and associated health behaviors: results from a population-based survey

**DOI:** 10.1186/1479-5868-7-19

**Published:** 2010-03-03

**Authors:** Catharine Wang, Elliot J Coups

**Affiliations:** 1 Boston University School of Public Health, Boston MA, USA; 2 The Cancer Institute of New Jersey, New Brunswick NJ, USA; 3 UMDNJ-Robert Wood Johnson Medical School, New Brunswick NJ, USA

## Abstract

**Background:**

Several genetic variants are associated with obesity risk. Promoting the notion of genes as a cause for obesity may increase genetically deterministic beliefs and decrease motivation to engage in healthy lifestyle behaviors. Little is known about whether causal beliefs about obesity are associated with lifestyle behaviors. Study objectives were as follows: 1) to document the prevalence of various causal beliefs about obesity (i.e., genes versus lifestyle behaviors), and 2) to determine the association between obesity causal beliefs and self-reported dietary and physical activity behaviors.

**Methods:**

The study data were drawn from the 2007 Health Information National Trends Survey (HINTS). A total of 3,534 individuals were included in the present study.

**Results:**

Overall, 72% of respondents endorsed the belief that lifestyle behaviors have 'a lot' to do with causing obesity, whereas 19% indicated that inheritance has 'a lot' to do with causing obesity. Multinomial logistic regression analyses indicated that the belief that obesity is inherited was associated with lower reported levels of physical activity (OR = 0.87, 95% CI: 0.77-0.99) and fruit and vegetable consumption (OR = 0.87, 95% CI: 0.76-0.99). In contrast, the belief that obesity is caused by lifestyle behaviors was associated with greater reported levels of physical activity (OR = 1.29, 95% CI: 1.03-1.62), but was not associated with fruit and vegetable intake (OR = 1.07, 95% CI: 0.90-1.28).

**Conclusions:**

Causal beliefs about obesity are associated with some lifestyle behaviors. Additional research is needed to determine whether promoting awareness of the genetic determinants of obesity will decrease the extent to which individuals will engage in the lifestyle behaviors essential to healthy weight management.

## Introduction

The causes of obesity are multifactorial [1]. Recent evidence has begun to shed light on the genetic contributions to obesity [2]. In the 2005 update of the human obesity gene map, 127 candidate genes for obesity were reported, 22 of which were replicated with multiple populations [3]. The majority of rare monogenic or single-gene disorders related to obesity appear to function via the regulation of appetite and impairment of satiety rather than the regulation of metabolism [2,4,5]. Common obesity, however, is mostly polygenic and due to the contribution of multiple genes and genetic variants.

Advances in obesity genetics, as well as the genetics of common diseases, has led to a proliferation of direct-to-consumer marketing of genetic tests that enable the identification of one's genetic risk for these conditions. It has been argued that such testing is premature, given the state of the science, and concerns have been raised about the clinical utility of this type of risk information [6-8]. Currently, it is unknown whether providing individuals with information about the genetic determinants of obesity will motivate or discourage weight management behaviors. Researchers have postulated that the provision of genetic information will influence both the cognitive representations or beliefs that people hold about the causes of a disease, as well as their beliefs about the solutions to reducing risk for disease [9,10]. Consequently, genetic risk information for obesity could have negative implications if providing feedback increased people's beliefs about the role of genetics in obesity, but decreased their beliefs about the efficacy and importance of lifestyle behaviors to manage obesity risk. Underlying this notion is the possibility that communicating the genetic risks of obesity might inadvertently serve to reinforce genetically deterministic thinking, which may reduce individuals' engagement in lifestyle behaviors important in maintaining a healthy weight.

As a first step to understanding the possible implications of communicating obesity-related genetic information, this study was undertaken with two main objectives. First, we set out to document the prevalence and correlates of causal beliefs about obesity (i.e., genes versus lifestyle behaviors) in a population-based sample in the United States. Second, we sought to determine the association between obesity causal beliefs and self-reported dietary and physical activity behaviors. The results of this study can shed light on whether obesity causal beliefs might function as a potential mechanism by which the provision of information about the genetic determinants of obesity influences behaviors important in healthy weight management.

## Methods and Procedures

### Procedure

The study data were drawn from the 2007 Health Information National Trends Survey (HINTS), a national probability survey of health information among U.S. adults conducted by the National Cancer Institute. Participants in the 2007 HINTS were recruited via random digit dialing or a mailing sent to a random sample of U.S. addresses. Participants completed a one-time telephone or paper and pencil survey. Some survey questions differed between the two modes of administration. For the purposes of the current study, we only used data from the mailed paper and pencil survey, as it utilized a two-item cups measure of fruit and vegetable intake that may have greater validity than the two-item servings measure used in the telephone survey (Amy Yaroch, PhD, personal communication, March 2009). The household response rate to the mailed survey was 44.0% and the within-household response rate was 77.4%. The 2007 HINTS utilized a cross-sectional, complex sample survey design, data weighting, and jackknife variance estimation. Further information about the HINTS is available elsewhere [11].

### Participants

A total of 7,674 individuals were recruited to the 2007 HINTS, 3,582 of whom completed the survey via mail. We excluded 48 individuals who were missing data for both of the obesity causal belief items, leaving an available sample size of *N *= 3,534.

### Measures

#### Demographics

Participants indicated their sex, age, race/ethnicity, and level of education. They also reported their height and weight from which we calculated body mass index (BMI) and categorized individuals as being not overweight/obese, overweight, or obese [12].

#### Obesity causal beliefs

Participants answered two questions about the causes of obesity: "To what extent do you believe that obesity is inherited?"; and "To what extent do you believe that obesity is caused by overeating and not exercising?". Each item was answered on a 4-point Likert-type scale from *not at all *to *a lot*. The correlation between the items was very low (*r *= -.05, *p *= .002), justifying their separate examination in the analyses. Responses to the second question were strongly negatively skewed, with 72.4% of participants answering *a lot*. Thus, we dichotomized responses to this question to denote individuals who answered *a lot *versus those who did not.

#### Physical activity

Participants indicated how often each week, and for how long, they engage in physical activity or exercise of at least moderate intensity, which was defined as activities that make you breathe somewhat harder than normal (e.g., brisk walking, cycling at a regular pace, heavy gardening). Based on national guidelines [13], we categorized individuals into three groups according to their total amount of weekly activity of at least moderate intensity: (a) sedentary - no weekly activity; (b) insufficient activity - engage in some activity but less than 150 minutes per week; (c) met guidelines - engage in at least 150 minutes of activity per week.

#### Fruit and vegetable intake

Participants read examples of the amount of fruits and vegetables that count as 1 cup (e.g., 1 small apple, 8 large strawberries, 12 baby carrots, 1 medium potato). Using 7 response options from *none *to *4 cups or more*, they then indicated approximately how many cups of fruit and how many cups of vegetables they consume each day. We summed responses for the two items to create an index of daily fruit/vegetable intake (with higher scores representing higher intake).

### Statistical Analysis

Initial regression analyses were conducted to examine demographic correlates of the two obesity causal belief items. In a multiple linear regression analysis, the demographic characteristics (sex, age, race/ethnicity, education level, and BMI) were included as independent variables with the inherited causal belief item as the dependent variable. In a multiple logistic regression analysis, the demographic characteristics were included as independent variables with the lifestyle obesity causal belief item (i.e., obesity is due to overeating and not exercising) as the dichotomous dependent variable. Next, we conducted two multinomial logistic regression analyses to examine correlates of physical activity and fruit/vegetable intake. In each analysis, the independent variables were the demographic and obesity causal belief items and the ordinal dependent variable was physical activity or fruit/vegetable intake. All statistical analyses were conducted using SUDAAN software (version 9.0.1; Research Triangle Institute, Research Triangle Park, NC). All percentages reported in the Results section are weighted and all sample sizes are unweighted. A cutoff of *p *< .05 was used to determine statistical significance for all analyses.

## Results

### Sample Characteristics

Descriptive statistics regarding the demographic and causal belief items are shown in Table 1. There was considerable variability with regard to the age, race/ethnicity, and level of education of the study participants. More than a quarter of the participants were obese and a third were overweight. Less than a quarter of participants indicated that inheritance has 'a lot' to do with causing obesity. Over two-thirds of participants indicated that they believe obesity is caused 'a lot' by overeating and not exercising.

**Table 1:** Descriptive Statistics for the Demographic Characteristics and Obesity Causal Belief Items

	Unweighted Sample Size (*n*)	Weighted Sample %
Sex		
Male	1372	48.9
Female	2159	51.1
Missing (*n*)	3	
		
Age (years) (*M *= 45.6)		
18-39	860	39.8
40-49	644	20.4
50-59	809	17.0
60-69	603	10.8
≥ 70	573	12.0
Missing (*n*)	45	
		
Race/ethnicity		
Hispanic	309	12.5
Non-Hispanic white	2460	69.5
Non-Hispanic black	431	11.5
Non-Hispanic other	225	6.5
Missing (*n*)	109	
		
Education		
Less than high school	306	13.8
High school graduate	805	24.4
Some college	1130	36.8
College graduate	1269	25.0
Missing (*n*)	24	
		
Body mass index (*M *= 27.6)		
Not overweight/obese (BMI < 25 kg/m^2^)	1286	37.3
Overweight (BMI 25-29.9 kg/m^2^)	1168	34.0
Obese (BMI ≥ 30 kg/m^2^)	1018	28.7
Missing (*n*)	62	
Belief that obesity is inherited		
Not at all	334	9.7
A little	890	25.2
Some	1670	46.4
A lot	627	18.6
Missing (*n*)	13	
		
Belief that obesity is caused by overeating and not exercising		
Not at all		
A little	30	0.8
Some	132	4.0
A lot	817	22.8
Missing (*n*)	2542	72.4
	13	

*Note*. *N *= 3,534. Data are from respondents who completed a paper and pencil mailed survey for the 2007 Health Information National Trends Survey (HINTS).

### Correlates of Obesity Causal Beliefs

The results of multiple regression analyses examining correlates of obesity causal beliefs are shown in Table 2. Age, race/ethnicity, and education were not associated with the belief that obesity is inherited. There was a marginally significant association (*p *= .056) suggesting that women were more likely to believe that obesity is inherited than men. Individuals who were obese more strongly endorsed the belief that obesity is inherited than individuals who were not overweight or obese.

**Table 2:** Results of Multiple Regression Analyses Examining Correlates of Obesity Causal Beliefs

	Belief that Obesity is Inherited		Belief that Obesity is Caused by Overeating and Not Exercising	
		
	*b*(95% CI)	*p *value^a^	OR (95% CI)	*p *value^a^
Sex	-0.09 (-0.18, 0.00)	.056	0.90 (0.75, 1.08)	0.247
Age (years)		.098		0.153
18-39	Ref		Ref	
40-49	0.05 (-0.06, 0.16)	.385	0.78 (0.57, 1.07)	
50-59	0.04 (-0.07, 0.15)	.486	0.83 (0.61, 1.13)	
60-69	-0.03 (-0.14, 0.09)	.62	1.03 (0.76, 1.39)	
≥ 70	-0.13 (-0.28, 0.02)	.09	0.99 (0.69, 1.42)	
				
Race/ethnicity		.752		0.002
Hispanic	-0.01 (-0.22, 0.20)	.91	1.61 (1.02, 2.54)	
Non-Hispanic white	Ref		Ref	
Non-Hispanic black	0.01 (-0.12, 0.14)	.887	0.65 (0.46, 0.91)	
Non-Hispanic other	0.12 (-0.10, 0.34)	.292	1.18 (0.76, 1.83)	
				
Education		.182		0.019
Less than high school	0.07 (-0.09, 0.23)	.373	0.96 (0.60, 1.54)	
High school graduate	0.10 (0.00, 0.20)	.056	0.67 (0.51, 0.88)	
Some college	0.08 (-0.01, 0.17)	.071	0.98 (0.76, 1.24)	
College graduate	Ref		Ref	
				
Body mass index		.007		0.404
Not overweight/obese	Ref		Ref	
Overweight	0.05 (-0.05, 0.16)	.334	1.17 (0.92, 1.50)	
Obese	0.19 (0.07, 0.31)	.002	1.15 (0.85, 1.56)	

*Note*. Data are from respondents who completed a paper and pencil mailed survey for the 2007 Health Information National Trends Survey (HINTS).

^a ^The *p *values adjacent to each variable name (sex, age, race/ethnicity, and so on) represent the association between the variable and the obesity causal belief item.

Sex, age, and BMI were not associated with the belief that obesity is caused by overeating and not exercising. Individuals with a college degree were more likely to believe that obesity is caused by overeating and not exercising compared to those with a high school education. Compared to non-Hispanic white individuals, Hispanic individuals were more likely to believe that obesity is caused by overeating and not exercising and blacks were less likely to hold that belief.

### Correlates of Physical Activity and Fruit/Vegetable Intake

Just over a third of the participants (37.9%) were categorized as sedentary, 26.8% engaged in insufficient activity, and 35.3% met physical activity guidelines. Table 3 shows the results of the multinomial regression analysis examining correlates of physical activity. Demographic factors associated with engaging in a lower amount of physical activity included being female, older, having a lower education level, and being obese. Additionally, the belief that obesity is inherited was associated with lower reported levels of physical activity. The belief that obesity is caused by overeating and not exercising was associated with greater reported levels of physical activity. We also conducted exploratory analyses to examine whether there was a significant interaction between body mass index and each of the causal beliefs items. The presence of a statistically significant interaction would indicate that the association between the causal belief and physical activity varied according to body mass index. However, neither of the two interaction terms was statistically significant (*p*s > .309).

**Table 3:** Results of Multiple Multinomial Logistic Regression Analyses Examining Correlates of Physical Activity and Fruit and Vegetable Intake

	Physical Activity		Fruit and Vegetable Intake	
		
	OR (95% CI)	*p *value^a^	OR (95% CI)	*p *value^a^
Sex	1.31 (1.08, 1.59)	.006	0.71 (0.58, 0.85)	< .001
Age (years)		.009		.039
18-39	Ref		Ref	
40-49	0.81 (0.62, 1.06)		0.83 (0.64, 1.06)	
50-59	1.01 (0.77, 1.31)		0.96 (0.76, 1.20)	
60-69	0.73 (0.56, 0.96)		1.12 (0.88, 1.42)	
≥ 70	0.63 (0.45, 0.89)		1.21 (0.93, 1.57)	
				
Race/ethnicity		.113		.912
Hispanic	0.72 (0.47, 1.11)		0.98 (0.63, 1.52)	
Non-Hispanic white	Ref		Ref	
Non-Hispanic black	0.74 (0.53, 1.03)		1.09 (0.80, 1.50)	
Non-Hispanic other	0.79 (0.53, 1.18)		1.12 (0.68, 1.87)	
				
Education		< .001		< .001
Less than high school	0.39 (0.24, 0.62)		0.39 (0.24, 0.62)	
High school graduate	0.45 (0.35, 0.56)		0.45 (0.35, 0.56)	
Some college	0.84 (0.67, 1.05)		0.84 (0.67, 1.05)	
College graduate	Ref		Ref	
				
Body mass index		< .001		.016
Not overweight/obese	Ref		Ref	
Overweight	0.99 (0.75, 1.30)		0.88 (0.70, 1.10)	
Obese	0.50 (0.39, 0.64)		0.73 (0.59, 0.91)	
				
Belief that obesity is inherited	0.87 (0.77, 0.99)	.027	0.87 (0.76, 0.99)	.028
				
Belief that obesity is caused by overeating and not exercising	1.29 (1.03, 1.62)	.025	1.07 (0.90, 1.28)	.432

*Note*. Data are from respondents who completed a paper and pencil mailed survey for the 2007 Health Information National Trends Survey (HINTS).

^a ^The *p *values adjacent to each variable name (sex, age, race/ethnicity, and so on) represent the association between the variable and physical activity or fruit and vegetable intake.

Participants reported consuming a median of 3-5 cups of fruits and vegetables per day. As shown in Table 3, demographic factors associated with a lower fruit and vegetable intake included being male, younger, having a lower education level, and being obese. The belief that obesity is inherited was associated with lower fruit and vegetable consumption. The belief that obesity is caused by overeating and not exercising was not associated with fruit and vegetable intake. We again conducted exploratory analyses to examine whether there was a significant interaction between body mass index and each of the causal beliefs items. Neither of the two interaction terms was statistically significant (*p*s > .365).

## Discussion

To our knowledge, this is one of the first studies to examine whether causal beliefs about obesity are associated with health behaviors important in weight management. This study contributes to the growing literature examining causal beliefs about obesity among individuals in the general population [14,15] and highlights how public health messages about the genetic etiology of obesity may influence obesity-related causal beliefs, which in turn, may influence health behaviors.

Overall, the majority of individuals in the current study believed that overeating and inactivity contribute to obesity. Substantially fewer endorsed the notion that obesity is caused by inheritance and genetics. This finding is opposite to what is commonly reported by obese individuals [16] and suggests that non-obese individuals are more likely to ascribe to the belief that obesity can be controlled via lifestyle behaviors. Within the present study, greater BMI was positively associated with beliefs about the role of inheritance, suggesting that beliefs about the role of inheritance vary significantly between obese and non-obese individuals. Similar to findings among obese individuals, there was some evidence in the present study (*p *= .056) that beliefs about the role of inheritance in obesity may be more common among women than men [16].

This study provides evidence of the association between obesity causal beliefs and health behaviors essential in weight management. Beliefs about the role of inheritance in obesity were negatively associated with both self-reported physical activity and dietary intake. Beliefs about the role of lifestyle in obesity correlated in the opposite direction with physical activity, providing some support for the importance of emphasizing the role of health behaviors in the prevention and management of obesity. The lack of association between lifestyle beliefs and self-reported diet may be, in part, due to how the former was assessed. The belief about lifestyle as a cause of obesity was assessed with regard to "overeating and not exercising", whereas diet was assessed in terms of fruit and vegetable intake as opposed to behaviors related to overeating (e.g., caloric intake). Other research assessing causal beliefs in the context of cancer suggests that people distinguish between various causes, highlighting the importance of specificity in the measurement of causal beliefs [17].

Identifying the relationship between causal beliefs about obesity and health behaviors is important in order to understand the potential implications of communicating genetic risk information. More research is necessary to shed light on the mechanism by which causal beliefs might influence health behaviors. For example, beliefs about the heritability of obesity may correspond to greater fatalistic perceptions and lower perceptions of disease controllability. Available qualitative, hypothetical, and clinical studies have both supported [18-22] and refuted [16,23-25] this possibility. It has also been suggested that the provision of genetic risk information may influence beliefs about the efficacy of various responses or solutions, such as a healthy diet, to reduce disease risk [26-29]. Additional research, guided by strong theoretical foundations (e.g., attribution theory, self-regulation theory) will contribute greatly to our understanding of the impact of genetic risk information on behavioral outcomes [9,21].

Endorsement of the heritability of obesity may reflect either a sense of genetic determinism (i.e., disease is inevitable) or a sense of genetic susceptibility (i.e., increased disease risk that lifestyle changes can counteract). In the current study, 73% of individuals who indicated that inherited factors have "a lot" to do with causing obesity also said that it is caused "a lot" by overeating and lack of exercise. Prior research suggests that people have a complex understanding of the role of genetics in common diseases and attribute these diseases to both genes and behavior [15,30-32]. Future research is needed to further explore potential attribution profiles among those who endorse genetics or inheritance as a cause of obesity, since they may be associated with different behavioral outcomes.

The study findings suggest that causal beliefs and attributions are important to consider when communicating messages about disease risk factors. Notably, casual beliefs appear to vary across diseases [27,30,32]. Less than a quarter of individuals in this study believed that inheritance was important in obesity causation. Greater endorsement of the causal role of heredity has been reported for other diseases, such as breast and colorectal cancer, wherein over 75% of women believe it to be a cause [17]. The extent to which common diseases are viewed as "genetic" or "heritable" in nature appears to be illness-specific and this may have implications for how genetic risk information is processed, understood, and acted upon by individuals [9,33].

Research is needed to determine how prior casual beliefs and attributions influence individual responses to genetic risk messages. Related, studies are also needed on the extent to which causal beliefs can be modified. Initial evidence suggests that beliefs about the role of genetics in obesity can be altered [27,34,35]. Less is known about the implications of modifying causal beliefs and whether changing beliefs about the role of inheritance/heredity in obesity can occur without negatively influencing motivation to engage in weight management behaviors.

This study had several limitations. As part of a national population-based survey, we were limited by the use of single items to assess causal beliefs. Although this has been a common approach in prior research, we acknowledge the inherent limitations of single-item measures. Further research is needed on the measurement of disease causal beliefs [36]. In addition, assessments of physical activity and diet were based on self-reports, which may result in overestimates of actual behavior, but would not be expected to influence the association between beliefs and behaviors. Physical activity and fruit and vegetable intake were assessed using brief measures, although there is evidence for the acceptable psychometric properties of such measures [37-42]. Fruit and vegetable intake are the only dietary behaviors assessed in the 2007 HINTS. Future research should examine the association between obesity causal beliefs and other dietary behaviors (such as caloric intake and portion sizes). Finally, this study was cross-sectional in nature and inferences about causality cannot be made. Longitudinal studies are critically needed to determine the impact of modifying causal beliefs on behavioral outcomes of interest.

## Conclusions

Beliefs about the causes of obesity are associated with physical activity and dietary behaviors of individuals in the general population. Additional research is needed to determine whether promoting awareness of the genetic determinants of obesity will decrease the extent to which individuals will engage in the lifestyle behaviors essential to healthy weight management.

## Competing interests

The authors declare that they have no competing interests.

## Authors' contributions

CW conceived of the study, participated in the design of the study, and drafted the manuscript. EJC participated in the design of the study, performed the statistical analysis, and drafted the manuscript. Both authors reviewed, revised, and approved the final manuscript.
